# Impact of the pandemic on leisure physical activity and alcohol consumption

**DOI:** 10.1186/s12889-024-19100-w

**Published:** 2024-06-13

**Authors:** Fredrik Granström, Marika Wenemark, Karin Festin, Elin Good, Helena Frielingsdorf, Mats Lowén, Ingrid Rystedt

**Affiliations:** 1 Department of Health, Medicine and Caring Sciences, Linköping University, Linköping, Sweden; 2 Department of Cardiology, Linköping University, Linköping, Sweden; 3 Department of Acute Internal Medicine and Geriatrics, Linköping University, Linköping, Sweden

**Keywords:** COVID-19, Leisure physical activity, Alcohol consumption, Age, Sex, Socioeconomic status, Longitudinal, Questionnaire

## Abstract

**Background:**

The COVID-19 pandemic precipitated heightened morbidity and elevated mortality attributed to the SARS-CoV-2 infection. The pandemic also influenced health behaviors such as physical activity (PA) and alcohol consumption. The aim of this study was to examine changes in leisure PA and alcohol consumption in Sweden during the pandemic, and elucidate potential discrepancies in changes across demographic strata and socioeconomic status (SES).

**Methods:**

Data were retrieved from two waves of the longitudinal cohort study Life conditions, Stress and Health (LSH) (*n* = 2,523). Two measures of change were used; longitudinal change relative to baseline (2012–2015) and reported change compared to before the pandemic. For these two change measures, differences between sex, age group and SES were analyzed using multinomial logistic regression.

**Results:**

Regardless of the change measure, the proportion of individuals with diminished PA was notably higher among females compared to males. Furthermore, relative to baseline, females were less likely to have increased their PA, however according to the reported change they were more likely to have increased PA. Longitudinal change in PA compared to baseline followed a reversed age gradient, while, according to reported change, a decrease in PA during the pandemic was most prevalent in respondents 45 years of age at baseline (OR = 1.8, CI: 1.2–2.5) and respondents 50 years of age at baseline (OR = 1.7, CI: 1.2–2.4). High SES was associated with a greater variability in PA. Alcohol consumption was generally reduced during the pandemic. However, individuals aged 40 or 45 years at baseline were more likely than others to have initiated risky alcohol consumption.

**Conclusions:**

Females exhibited a greater propensity to alter their PA levels during the pandemic, with the most profound decreases observed among individuals of working ages. Despite a general downturn in alcohol consumption, individuals aged 40 and 45 had a heightened likelihood of having initiated risky alcohol consumption compared to individuals in other age cohorts. In conclusion, societal restrictions during a pandemic render a dual impact on PA levels. While posing a risk for decreased PA among individuals in working ages, the restrictions also present a potential window of opportunity to increase PA, particularly among females.

**Supplementary Information:**

The online version contains supplementary material available at 10.1186/s12889-024-19100-w.

## Background

The outbreak of the COVID-19 pandemic in early 2020 had a tremendous direct effect on population health, with high morbidity and mortality due to the Sars-Cov-2 virus. The pandemic was accompanied by confinements and recommendations introduced by governments and authorities to prevent the spread of infection. Moreover, to safeguard their health, a considerable share of the population, particularly elderly and individuals in medically vulnerable groups, opted for heightened levels of self-isolation beyond the recommendations issued by authorities. These changed life circumstances posed risks of indirect effects of the COVID-19 pandemic, e.g., social isolation, job loss, financial difficulties, stress and anxiety [1]. These indirect effects could also contribute to changes in health behaviors, e.g., leisure physical activity (PA) and alcohol consumption. During most of the pandemic, working from home, when possible, was mandatory or recommended in most countries. Studies have identified an increased risk of physical inactivity related to the transition to home working [2, 3]. Also, many fitness centers and sport centers were placed in lockdown, resulting in reduced opportunities for maintaining existing PA habits. Similarly, patterns of alcohol consumption were affected by the pandemic. A majority of restaurants and event venues which normally serve alcohol were closed, while most private social arrangements were prohibited. Consequently, availability of alcohol beverages in public settings was dramatically reduced, and alcohol consumption was thereby transferred to the privacy of the home environments. While this restricted socially induced drinking, it is worrisome considering that studies have shown that solitary drinking sometimes involves higher alcohol consumption than social drinking [4]. Furthermore, stressful life experiences are frequently associated with excessive alcohol consumption [5], and societal crisis situations tend to similarly increase alcohol consumption, especially among males, young people and those living in single households [6].

It is important to be aware of systematic changes in PA or alcohol consumption, on a population level, as both physical inactivity and excessive alcohol consumption are major risk factors for poor health. Physical inactivity is highlighted as a major cause of chronic diseases [7], and alcohol is an important contributor to the global disease burden [8]. Also, both physical inactivity and alcohol consumption account for a substantial preventable premature mortality worldwide [9]. Recently, it w reported that even moderate alcohol consumption is indeed a risk factor for ill-health [10, 11].

A systematic review [12], as well as an international online survey covering countries all over the world, have consistently found that the pandemic led to an overall decrease in PA [13]. However, another review of studies examining the change in PA during to the pandemic reported mixed results [14]. Even though most included studies found decreases in PA, about one third reported either an increase or a mixed pattern. In regards to the overall change in alcohol consumption during the pandemic, two systematic reviews found mixed patterns with a slight overweight for studies reporting increases in consumption, compared to studies reporting decreases in consumption [12, 15].

Prior to the pandemic, it was well established that PA habits and alcohol consumption differ among population groups. In Sweden, such examples include the fact that physical inactivity increases by age, and that the proportion of physically inactive individuals is larger among individuals with low socioeconomic status (SES). In addition, in Sweden, self-reported risk-consumption of alcohol in Sweden is more common among males and less common among both males and females aged 65 years of age and older, compared to individuals in younger age groups [16]. It is therefore likely that the impact of the pandemic on PA and alcohol consumption, respectively, differs among population groups. However, international studies that have examined sex differences in change in PA or alcohol consumption [17–26] and across SES groups [15, 27–37] report heterogeneous results.

Above and beyond the contextual differences among countries, change can also be measured in several ways [38]. The variation in specific change measures may partially explain the disparity in findings concerning the impact of the pandemic on PA and alcohol consumption.

Compared to most countries, Sweden handled the pandemic in a relatively unique manner, as there were no total lockdowns. Measures were primarily in the form of recommendations, which opened for a larger extent of individual freedom, e.g. in terms of how strictly to interpret and adhere to recommendations. It is therefore possible that the pandemic had a different impact on PA and alcohol consumption in Sweden, compared to in many other countries. Some previous Swedish studies have explored how PA habits were affected by the pandemic. One cross-sectional study reported an overall decrease in PA after the onset of the pandemic in both males and females. The largest decreases were found in the youngest and oldest age groups [39]. However confirmatory findings from longitudinal studies are still lacking. Further, we have not found studies evaluating change in alcohol consumption across subsets of the Swedish during the pandemic.

Increased knowledge on how health inequalities are potentially exacerbated by a pandemic in terms of health behavior is important for professionals working with prevention and health promotion. This knowledge will facilitate the development of effective strategies, that concurrently reduce the spread of the infection and minimize negative indirect effects on health behaviors.

Therefore, it is urgent to evaluate the Swedish strategy of tackling the pandemic and resulting effects on health behaviors. Further, it is important to know whether potential effects vary across population groups. To make this evaluation more robust, and to elucidate potential nuances in results, this study analyzed both longitudinal measured change and cross-sectional reported change.

### AIM

This study examined changes in leisure PA and alcohol consumption among middle aged and elderly Swedish persons before and during the pandemic, by using both a longitudinal change measure and a reported change measure. In addition, the aim was to contrast the two change measures to further elucidate patterns across demographic strata and SES.

## Materials and methods

This study utilized data from two waves of the longitudinal cohort study Life conditions, Stress and Health (LSH). The study population included a random sample of persons aged 40, 45, 50, 55, 60, 65 and 70 years in two counties in south-eastern Sweden (Östergötland and Jönköping). Between 2012 and 2015, individuals in the two counties were invited by the county councils to their primary health care center for a health dialogue, where health behaviors and life conditions were discussed, and health-promoting changes were suggested. Participants who attended these health dialogues were invited to participate in a wave of the LSH study called LSH II, which for the present study constitutes our baseline data. In total, 28,702 citizens were invited to health dialogues, of which 12,164 (42%) accepted. Of those, 6,860 (56%) individuals (3,880 females and 2,980 males) also agreed to participate in the LSH II study. In total, LSH II participants responded three postal questionnaires, two as inventories prior to the health dialogue and one additional LSH-specific questionnaire.

During the pandemic, in January 2021, a postal follow-up questionnaire was distributed to LSH II respondents in one of the two counties (Östergötland) (*n* = 3,643), covering in part the same questions as the baseline questionnaires, with the addition of several pandemic-related questions. This data collection was completed in April 2021 after one postal reminder. The response rate for the COVID questionnaire was 73%, resulting in a sample of 2,523 persons, 1,493 females and 1,030 males.

### Indicators of SES

Educational level and disposable household income represented indicators of SES. For LSH II, this information was retrieved at baseline (2012–2015) from registry data.

Educational level was measured in terms of highest achieved education and was divided into five categories: ‘Only elementary school’; ‘Two-year of secondary school’; ‘Three-or-four-year of secondary school’; ‘University studies of maximum three years’; and ‘University degree more than 3 years’.

Household income was divided into quartiles based on total disposable household income after taking the number of household members into account.

The two most common ways of measuring change in self-reported assessments are either to make a longitudinal comparison of self-reported assessments from the same individual at two different time points (measured change or indirect measure of change) or to ask the individuals to self-estimate whether any change has occurred from one time point to another (reported change or direct measure of change). Evaluations have shown that results sometimes can differ substantially depending on the type of measure being utilized [38]. In the present study, we therefore use both types of measures of evaluating changes in leisure PA and changes in alcohol consumption.

### Leisure PA

Both in the baseline and in the COVID questionnaires, information on leisure PA was captured from the question: ‘How much do you exercise physically in your leisure time?’, with options: ‘Sedentary leisure time (You spend most leisure time reading, watching TV, needlework or other sedentary pursuits)’, ‘Moderate exercise (You walk, bike, do gardening or similar activities at least 4 hours a week)’, ‘Strenuous exercise (You jog, swim, do aerobics, go skiing, do heavy gardening or similar activities at least 2 hours a week)’ and ‘Intense exercise (You exercise heavily, corresponding to competitive sports, such as running, swimming, skiing or similar activities regularly and several times a week’. The two last response categories were combined into one category (vigorous exercise). Also, the COVID questionnaire included a question prompting respondents to assess how their PA habits had changed since before the pandemic, with a bipolar response scale: ‘Considerably more now’, ‘Somewhat more now’, ‘unchanged’, Somewhat less now’ and ‘Considerably less now’. In the analyses, the two first and the two last categories were combined, resulting in a three-level variable: ‘More’, ‘Unchanged’ and ‘Less’.

### Alcohol consumption

Information on the extent of risky pattern of alcohol consumption was assessed by two questions targeting how much alcohol the respondents typically drink in one week, and how often the respondents drink a defined large amount on one occasion. If respondents typically have more than 9 drinks a week (females)/14 drinks a week (males) or 4 drinks (females)/5 drinks (males) on one occasion, their alcohol consumption was classified as risky based on national guidelines from the Swedish National board of Health and Welfare at the time of the study [40]. These questions were identical at baseline and in the COVID questionnaire. In addition, the COVID questionnaire included a question prompting respondents to assess how their alcohol habits had changed compared to before the pandemic, with the options: ‘Drink considerably more now’, ‘Drink somewhat more now’, ‘unchanged’, ‘Drink somewhat less now’ and ‘Drink considerably less now’. In our analyses, the two first and the two last response categories were combined, resulting in a three-level variable: ‘More’, ‘Unchanged’ and ‘Less’.

### Potential confounders

Several factors, in addition to a pandemic, can induce changes in leisure PA or alcohol consumption. Both financial problems and change in cohabitation (separations or divorces) increase the risk for physical inactivity [41, 42] and increased alcohol intake [43]. In addition, severe health problems are likely related to specific health behaviors. Therefore, one option could have been to exclude individuals with severe health problems from the analyses. However, in order not to lose statistical power we instead conducted a sensitivity analysis of how our results were affected by an exclusion of individuals with severe health problems. The results remained practically unchanged. Consequently, so we decided to keep all individuals in the analyses, and the potential confounding variables for the associations under study included: severe health problems, change in cohabitation status and impact of the pandemic on private financial situation.

Respondents were assessed to have severe health problems if they in the baseline questionnaire reported severe problems due to any of the following physician diagnosed conditions: myocardial infarction or stroke, angina pectoris, cancer, chronic lung disease, rheumatoid arthritis, asthma or allergy, gastrointestinal disease, musculoskeletal disorders, neurological disease, renal disease or depression.

Change in cohabitation status was assessed by questions in both questionnaires targeting whether respondents were married/cohabiting with a partner or not. Impact of the pandemic on the private financial situation of respondents was assessed by a question in the COVID questionnaire asking: “What impact has the pandemic in total had on your financial situation?”, with options: ‘No impact’, ‘Very negatively’, Somewhat negatively’, ‘Somewhat positively’, and ‘Very positively’.

### Statistical analyses

Longitudinal and reported change of leisure PA and alcohol consumption, respectively, were used as outcome measures and tested for associations with sex, age and the two indicators of SES (educational level and disposable household income), respectively. Bivariate associations were tested by chi-square tests. Multinomial logistic regression analysis was used to estimate odds ratios (OR) with 95% confidence intervals (95% CI), to describe the associations adjusted for confounders.

This adjustment was made in two steps. First, the associations were adjusted for status at baseline in terms of extent of severe health problems, sex, age (in models including SES), leisure PA (in models including changes in PA), risky alcohol consumption (in models including changes in risky alcohol consumption) (Model I). In order to examine whether the observed differences in change could be explained by material/structural factors (change in cohabitation status and/or impact of the pandemic on the private financial situation), associations were in a second step further adjusted for these variables (Model II). As the impact of the material/structural factors could differ across demographic or SES groups, we also tested to include interaction effects between the material/structural factors and sex, age, and the SES variables, respectively. However, none of these interaction effects were statistically significant, so we opted to only include main effects in model II.

All analyses were performed using SPSS version 29 (IBM Corp, Armonk, New York, USA).

## Results

### Leisure PA

Overall, more than half of the respondents reported unchanged leisure PA levels during the pandemic. Among the remaining respondents, it was more common to report decreases in leisure PA than increases in leisure PA according to measured change (19.9% vs. 12.8%). Similar ratio was observed in analyses using reported change, although the actual percentages were almost 7% units higher (26.7% vs. 19.5%) (Table 1). More females than males decreased their leisure PA levels during the pandemic, according to both the longitudinal change measure and the reported change measure. The proportion of respondents with a longitudinal decrease in their leisure PA were higher in the oldest group (70 years of age at baseline) than in other age groups (Table 1). For both change measures, respondents 45 years of age at baseline and respondents 50 years of age at baseline more frequently decreased their leisure PA, compared to respondents in most other age groups (Table 1).

**Table 1:** Overview of change in leisure PA, longitudinal change and reported change

		Longitudinal change in leisure PA					Reported change in leisure PA				
		Unchanged	More PA	Less PA	Total	p-value	Unchanged	More PA	Less PA	Total	p-value
Total n (%)		1576 (67.3)	301 (12.8)	466 (19.9)	2343		1343 (53.8)	486 (19.5)	665 (26.7)	2494	
Sex	Males	654 (66.9)	148 (15.1)	176 (18.0)	978	0.007	635 (62.1)	170 (16.6)	218 (21.3)	1023	< 0.001
	Females	922 (67.5)	153 (11.2)	290 (21.2)	1365		708 (48.1)	316 (21.5)	447 (30.4)	1471	
Age	40	148 (63.5)	48 (20.6)	37 (15.9)	233	< 0.001	118 (47.4)	62 (24.9)	69 (27.7)	249	< 0.001
	45	168 (59.8)	42 (14.9)	71 (25.3)	281		141 (47.2)	62 (20.7)	96 (32.1)	299	
	50	174 (62.1)	39 (13.9)	67 (23.9)	280		150 (51.4)	47 (16.1)	95 (32.5)	292	
	55	226 (67.3)	44 (13.1)	66 (19.6)	336		180 (50.0)	69 (19.2)	111 (30.8)	360	
	60	282 (70.0)	57 (14.1)	64 (15.9)	403		228 (53.6)	106 (24.9)	91 (21.4)	425	
	65	307 (71.1)	51 (11.8)	74 (17.1)	432		277 (60.2)	81 (17.6)	102 (22.2)	460	
	70	271 (71.7)	20 (5.3)	87 (23.0)	378		249 (60.9)	59 (14.4)	101 (24.7)	409	
Educational level	Compulsory	187 (70.0)	31 (11.6)	49 (18.4)	267	0.18	186 (64.1)	49 (16.9)	55 (19.0)	290	< 0.001
	Secondary school 2 years	446 (68.6)	76 (11.7)	128 (19.7)	650		431 (63.2)	102 (15.0)	149 (21.8)	682	
	Secondary school 3 years	249 (70.3)	41 (11.6)	64 (18.1)	354		207 (55.1)	73 (19.4)	96 (25.5)	376	
	Post-secondary school 3 years	265 (62.2)	58 (13.6)	103 (24.2)	426		227 (49.2)	94 (20.4)	140 (30.4)	461	
	Post-secondary school more than 3 years	428 (66.6)	95 (14.8)	120 (18.7)	643		290 (42.6)	167 (24.6)	223 (32.8)	680	
Household income	Q1	395 (67.8)	76 (13.0)	112 (19.2)	583	0.90	369 (59.2)	99 (15.9)	155 (24.9)	623	0.002
	Q2	385 (65.5)	83 (14.1)	120 (20.4)	588		350 (55.7)	131 (20.9)	147 (23.4)	628	
	Q3	399 (67.6)	75 (12.7)	116 (19.7)	590		319 (51.6)	118 (19.1)	181 (29.3)	618	
	Q4	396 (68.3)	67 (11.6)	117 (20.2)	580		303 (48.8)	138 (22.2)	180 (29.0)	621	

For the reported change both more leisure PA and less leisure PA were more common among respondents with the highest educational level. Reports of unchanged leisure PA levels were more likely among respondents with lower educational levels. When using the longitudinal change measure, the magnitude of differences between SES groups regarding changes in leisure PA was smaller (Table 1).

The proportion of respondents with a longitudinal increase in their leisure PA was lower among females than among males. However, according to reported change the proportion of respondents with an increase in leisure PA was higher among females.

### Alcohol consumption

Alcohol consumption generally decreased during the pandemic, both in terms of a higher proportion of respondents reporting risky pattern of alcohol consumption at baseline but not during the pandemic (20.6%) than the other way round (7.6%), and according to reported change in alcohol consumption from before the pandemic (13.4% reporting a decrease vs. 8.7% reporting an increase) (Table 2). The reported change in alcohol consumption did not significantly differ between sexes. The proportion of respondents who increased their alcohol consumption during the pandemic was highest among those 40 years of age at baseline (46–49 years during the pandemic). This applied both the proportion of respondents that reported risky alcohol consumption during the pandemic, but not at baseline and the proportion estimating an increase in their alcohol consumption compared to before the pandemic (Table 2). The proportion of respondents with risky pattern of alcohol consumption during the pandemic, but not at baseline was also relatively high in the oldest group (70 years of age at baseline).

**Table 2:** Overview of change in alcohol consumption, longitudinal change and reported change

		Longitudinal change in risk consumption of alcohol					Reported change in alcohol consumption				
		Unchanged	Change from consumption not considered risky to risky consumption	Change from risky consumption to a consumption not considered risky	Total	p-value	Unchanged	Drinks more	Drinks less	Total	p-value
Total n (%)		1658 (71.8)	175 (7.6)	475 (20.6)	2307		1884 (77.9)	210 (8.7)	325 (13.4)	2419	
Sex	Males	654 (69.7)	63 (6.7)	220 (23.6)	937	< 0.001	766 (77.5)	93 (9.4)	130 (13.1)	989	0.56
	Females	1004 (73.3)	112 (8.2)	254 (18.5)	1370		1118 (78.2)	117 (8.2)	195 (13.6)	1430	
Age	40	160 (69.0)	23 (9.9)	49 (21.1)	232	< 0.001	163 (66.0)	35 (14.2)	49 (19.8)	247	0.002
	45	213 (75.0)	20 (7.0)	51 (18.0)	284		218 (73.6)	27 (9.1)	51 (17.2)	296	
	50	193 (72.6)	17 (6.4)	56 (21.1)	266		228 (80.0)	22 (7.7)	35 (12.3)	285	
	55	252 (75.4)	16 (4.8)	66 (19.8)	334		279 (80.2)	26 (7.5)	43 (12.4)	348	
	60	282 (71.2)	31 (7.8)	83 (21.0)	396		328 (79.2)	34 (8.2)	52 (12.6)	414	
	65	303 (71.3)	31 (7.3)	91 (21.4)	425		355 (80.9)	38 (8.7)	46 (10.5)	439	
	70	255 (68.9)	37 (10.0)	78 (21.1)	370		313 (80.3)	28 (7.2)	49 (12.6)	390	
Educational level	Compulsory	190 (73.9	23 (8.9)	44 (17.1)	257	0.04	221 (79.8)	22 (7.9)	34 (12.3)	277	< 0.001
	Secondary school 2 years	434 (69.7)	58 (9.3)	131 (21.0)	623		545 (83.3)	39 (6.0)	70 (10.7)	654	
	Secondary school 3 years	265 (75.3)	17 (4.8)	70 (19.9)	352		290 (79.7)	34 (9.3)	40 (11.0)	364	
	Post-secondary school 3 years	312 (72.1)	25 (5.8)	96 (22.2)	433		343 (76.2)	39 (8.7)	68 (15.1)	450	
	Post-secondary school more than 3 years	455 (71.3)	51 (8.0)	132 (20.7)	638		481 (71.9)	76 (11.4)	112 (16.7)	669	
Household income	Q1	381 (67.3)	62 (11.0)	123 (21.7)	566	< 0.001	468 (80.0)	42 (7.2)	75 (12.8)	585	0.25
	Q2	426 (74.2)	39 (6.8)	109 (19.0)	574		480 (78.9)	43 (7.1)	85 (14.0)	608	
	Q3	422 (73.9)	28 (4.9)	121 (21.2)	571		465 (76.1)	63 (10.3)	83 (13.6)	611	
	Q4	427 (72.0)	45 (7.6)	121 (20.4)	593		468 (76.6)	62 (10.1)	81 (13.3)	611	

Among SES groups, both the proportion of respondents reporting increased alcohol consumption and the proportion of respondents reporting decreased alcohol consumption were highest among respondents in the group with the highest educational level. According to the longitudinal change measure, the proportion of respondents changing from a pattern of alcohol consumption not determined as risky at baseline to a pattern of risky alcohol consumption during the pandemic was somewhat among respondents in higher in the lowest SES groups.

### Multivariate analyses

#### Leisure PA

**Fig. 1:**
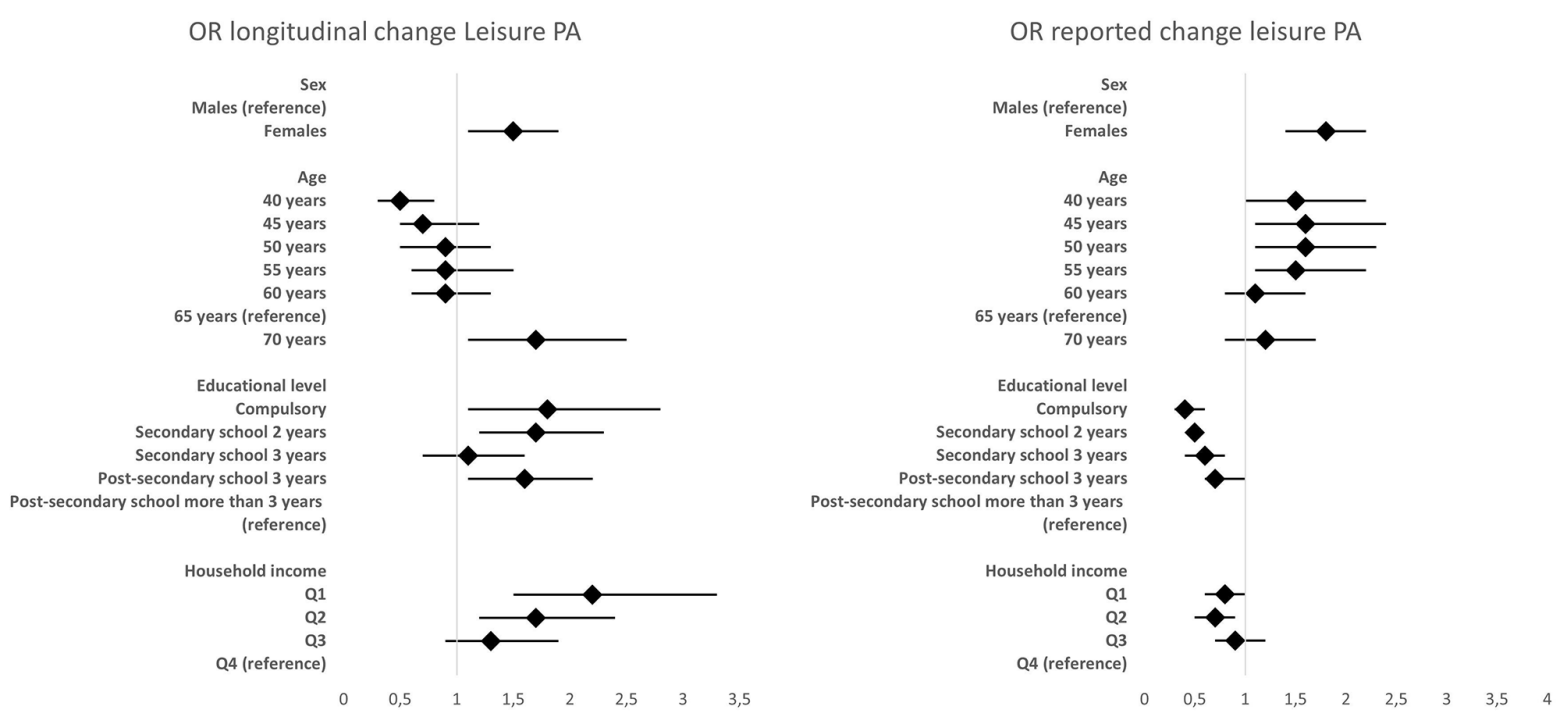
Results of logistic regression predicting a decrease in leisure PA by population groups. Legend: Odds ratios (OR) including 95% confidence intervals. Analyses were adjusted for sex, age (only for educational level and household income), baseline leisure PA and severe health problems

Figure 1 & additional files 1 & 3 illustrate that females were more likely to decrease their leisure PA in model I (after adjusting for baseline level of leisure PA and prevalence of severe health problems) (OR = 1.5, CI: 1.1–1.9 for the longitudinal change measure, OR = 1.8, CI: 1.4–2.2 for the reported change). This finding did not change in model II (further adjustments for change in cohabitation status and impact of the pandemic on the private financial situation).

In model I, the longitudinal change in leisure PA followed an age gradient (Fig. 1 & additional file 1). The older the respondents the smaller the proportion with an increase in their leisure PA levels and the larger the proportion with a decrease. This pattern remained in model II. The likelihood of a reported decrease in leisure PA was highest among respondents 45 years of age at baseline (OR = 1.6, CI: 1.1–2.4) and respondents 50 years of age at baseline (OR = 1.6, CI: 1.1–2.3).

In model I, respondents with the highest educational level were more likely to report an increase and a decrease in leisure PA, respectively, compared to respondents with other educational levels. This can be seen as the highest educational level was used as reference group, and the OR’s for all other education groups OR was below 1 (additional file 3). This finding was the same in model II. A similar pattern was found when using household income as an indicator of SES. Respondents in the highest income quartile were most likely either to have increased or decreased their leisure PA levels. However, these associations were not as strong as the associations in the analyses utilizing educational level as proxy for SES, and many differences were not statistically significant. In the bivariate analyses of the associations between measures of SES and the longitudinal change in leisure PA, no significant differences were found (Table 1). However, at baseline, respondents in the lower SES groups were considerably more likely to report being physically inactive in their leisure time than respondents in higher SES groups (additional file 5). This meant that for a larger proportion of respondents in the lower SES groups a measured decrease was technically impossible, as they already belonged to the lowest category at baseline, compared to respondents in higher SES groups. In model I, all other education level groups, except for the one with the highest secondary education, were more likely to have had a longitudinal decrease in their leisure PA levels compared to the reference group (post-secondary education longer than 3 years) (Fig. 1 & additional file 1).

In model I, the likelihood of an increase in leisure PA levels were smaller among females compared to males according to the longitudinal change measure (OR = 0.7, CI: 0.5–0.9), but higher among females according to the reported change measure (OR = 1.6, CI: 1.3-2.0) (additional files 1 & 3). The proportion of respondents with a reported increase in their leisure PA was highest among those 40 years of age at baseline (OR = 1.7, CI: 1.1–2.6), 45 years of age at baseline (OR = 1.6, CI: 1.1–2.4), and 60 years of age at baseline (OR = 1.6, CI: 1.1–2.2).

### Alcohol consumption

**Fig. 2:**
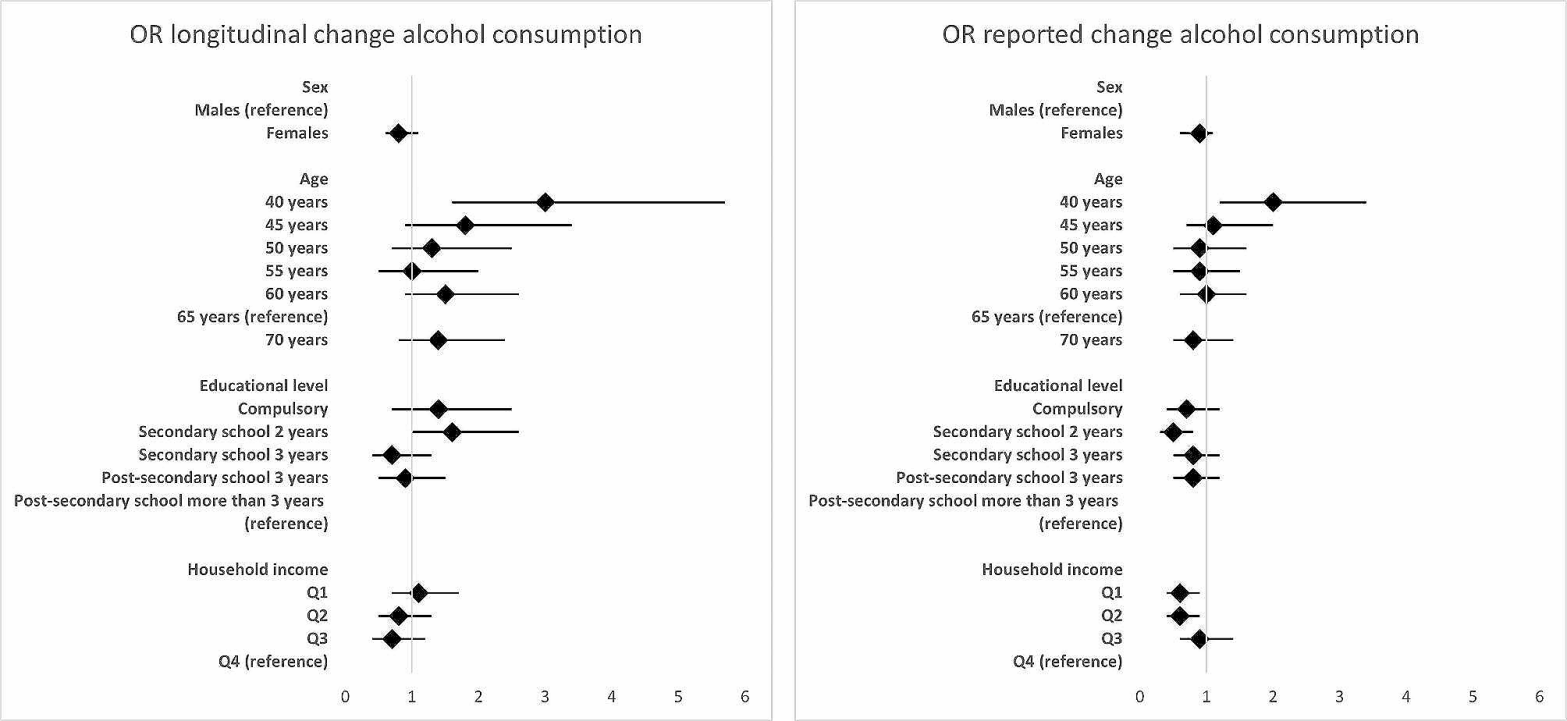
Results of logistic regression predicting an increase in alcohol consumption by population groups. Legend: Odds ratios (OR) including 95% confidence intervals. Analyses were adjusted for sex, age (only for educational level and household income) and severe health problems

There were no differences between males and females in the likelihood of having changed from a pattern of alcohol consumption not considered risky at baseline to a pattern considered risky during the pandemic. Likewise, the reported change in alcohol consumption did not differ significantly between males and females (Fig. 2).

In model I, the likelihood of a change into a pattern of risky alcohol consumption was somewhat elevated for respondents aged 70 years at baseline, but not statistically significant (OR = 1.5, OR: 0.9–2.6) (Fig. 2 & additional file 2). On the other hand, respondents 40 years of age at baseline were more likely to have increased their alcohol consumption, compared to the reference group (60 years of age) (Fig. 2). This result was found both in terms of longitudinal change (OR = 3.0, CI: 1.6–5.7) and reported change (OR = 2.0, CI: 1.2–3.4). This higher likelihood of an increase in alcohol consumption was partly explained by material/structural factors, as the OR decreased somewhat in model II (additional file 2). Furthermore, according to the longitudinal change measure, respondents 45 years old at baseline had a higher probability of having changed their drinking habits to a pattern of risky alcohol consumption during the pandemic, however not statistically significantly higher than the reference group (60 years of age) (OR = 1.8, CI: 0.9–3.4) (Fig. 2).

The differences in the proportion of respondents having moved to a risky pattern of alcohol consumption across SES groups were small both in model I and model II, measured with longitudinal change (Fig. 2 and additional file 2). Respondents with the shortest secondary education (2 years) were most likely to have changed their behavior towards a pattern of risky alcohol consumption, with a borderline statistical significance (OR = 1.6, CI: 1.0-2.6) compared to the reference group (post-secondary education of more than 3 years). A reported increase in alcohol consumption or a reported decrease in alcohol consumption, respectively, were more common among respondents in the highest SES groups (additional file 4). However, this circumstance only applies to the two response categories ‘Drink somewhat more’ and ‘Drink somewhat less’. For the more extreme changes: ‘Drink considerably more’ and ‘Drink considerably less’, the proportions of these responses were not larger among respondents in the highest SES groups, compared to respondents in other SES groups.

## Discussion

Triangulating two different measures of change, a longitudinal change measure and a reported change measure, we showed that the extent of leisure PA was considerably affected by the pandemic across several population groups. Both increases and decreases in leisure PA levels were relatively common. Furthermore, we observed a general decrease in alcohol consumption, which was persistent across population groups.

Both according to the longitudinal change measure and the reported change measure, females were more likely than males to decrease their leisure PA levels after the onset of the pandemic. Most other studies on changes in PA during the pandemic have reported that decreases in PA were more common among males [18–21, 44]. However, similarly to this study, a British study found that females were more likely to decrease their levels of PA [17]. On the other hand, based on reported change, females were also more likely to increase their PA, a result which is in line with results from a UK study [44]. We also found that both longitudinal change and reported change indicate that younger respondents (40 or 45 years old at baseline) were more likely to increase their leisure PA levels. According to the longitudinal change measure, the likelihood of a reduction in leisure PA during the pandemic increased by age. However, according to the reported change, respondents 55 years or younger at baseline were more likely to report a decrease in leisure PA compared to respondents older than 55 years. As 6–9 years had passed from baseline of LSH II to the outbreak of the pandemic, the majority of respondents 55 years or younger at baseline were likely to still be in the work force during the pandemic, while the majority of the respondents above 55 at baseline were probably retired during the pandemic. The finding that younger respondents are more likely to increase their leisure PA levels and older respondents more likely to decrease their leisure PA can potentially be viewed as a natural aging process. However, our opposite finding, based on the reported change, that persons in working ages more frequently decreased their leisure PA aligns well with the result of a Polish study [20]. Many employees in Sweden worked from home during the time period during pandemic captured in the present study. Consequently, our findings echo previous studies suggesting an association between working from home and physical inactivity [2, 3]. One explanation may be that walking or travelling by bike to and from work constituted regular PA before the pandemic, and after the transition to working from home this type of PA disappeared. However, the majority of respondents 55 years or younger at baseline reported still mainly working from their regular workplace when responding the questionnaire. Only 25% of them worked primarily from home. With the reported change measure, a divergent finding in the present study relative to many previous studies [39, 45–47] was that the oldest respondents did not decrease their leisure PA levels more than respondents of other ages. This might be explained by an inability in some of the other studies to separate the overall trend of larger decrease in PA in older age groups from the specific pandemic-related change in PA.

Several previous studies have reported an increased SES gradient in PA during the pandemic [21–25, 48]. Results using the longitudinal change measure in the present study confirm this tendency, even though the time-period for the measurement in this study included several years before the pandemic. However, on the contrary, when analyzing reported change, specifically targeting change from the onset of the pandemic, respondents with the highest SES were most likely to decrease their leisure PA.

Regardless of change measure, we found a general reduction in alcohol consumption during the pandemic. This reduction was most profound among respondents 40 and 45 years old at baseline. However, among respondents 40 years of age at baseline, the pattern was heterogeneous, also including a higher likelihood of increased alcohol consumption. Our finding that alcohol consumption more frequently changed among respondents of middle age compared to older respondents is in line with results from several studies [31, 34–36, 49–51]. According to the longitudinal change measure, males were more likely to go from a pattern of risky alcohol consumption at baseline to a pattern of alcohol consumption not considered risky during the pandemic. However, the reported change in alcohol consumption during the pandemic did not differ between males and females. An interpretation of this result can be that the more profound reduction in alcohol consumption among males occurred well before the pandemic, and that the changes in alcohol pattern during the pandemic did not differ between males and females. Compared to respondents in other SES groups, respondents in higher SES groups both more often reported an increase in alcohol consumption and more often reported a decrease in alcohol consumption. Several previous studies have found a larger increase in alcohol consumption during the pandemic among those with high educational level [30, 33, 35, 36, 52, 53], and one study reported the same pattern as in the present study with both larger increases and larger decreases in alcohol consumption among respondents in higher education groups [44]. However, in the present study these changes were not seen when using the longitudinal change measure, also targeting a period well before the pandemic, and the reported changes were exclusively explained by a higher proportion reporting ‘somewhat more’ or ‘somewhat less’.

An interesting feature of this study was that change in health behaviors was measured in two ways, both using a longitudinal change measure and a reported change measure. Reported change can be affected by both recall bias [54] and present state effect [55]. However, recall bias is not a major problem when the recall time interval is relatively short and the specified time point to recall is meaningful to the respondent [38]. The time interval to recall in the present study was about a year, but as the outbreak of the pandemic was such an overwhelming shift in life circumstances for the respondents. Consequently, recall bias was probably only of minor importance. However, the results might well be biased by the present state effect. This effect implies that respondent´s assessments of change are affected by their current states. For instance, respondents currently physically inactive are more likely to report a decrease in leisure PA even though their leisure PA is unchanged. This means that reported changes often are overestimated [56]. On the other hand, a disadvantage and challenge using a longitudinal change measure is the fact that floor and ceiling effects can sometimes bias assessments [57]. This occurred in the present study, as respondents with leisure PA matching the lowest response category already at baseline did not have a response category matching having presently even less leisure PA. Another drawback with the use of a longitudinal change measure is that small changes are sometimes not possible to detect when using only a few response categories. There might be that respondents have decreased or increased their leisure PA level, but that the new level still falls into the same response category.

In addition to the methodological differences discussed above, an important aspect in this study is that the reported change captures a time interval of about a year, whereas the longitudinal change measure captures a time interval of 6–9 years. This means that the disparate findings when using the two types of measures can be explained either by the validation problems discussed above or by the fact that some changes in leisure PA and/or alcohol consumption took place before the onset of the pandemic and was therefore not relevant in the reported change measurement. For instance, as mentioned above, the results for our longitudinal change measure aligned well with findings in previous studies in that SES differences in PA increased over time, including after the outbreak of the pandemic. However, the reported change complemented this picture by showing that specifically after the onset of the pandemic respondents with high SES more often decreased their leisure PA than respondents with lower SES. The divergent findings in relation to most other studies can either be explained by the fact that those studies were not able to disentangle the specific pandemic effect from the long-term trend of increasing SES inequalities in PA, or that our results were influenced by a present state effect resulting in an overestimation of the reported decrease in PA among respondents with high SES. They might well have had an ambition to spend more time with leisure PA during the pandemic than they were able to, and therefore had an impression that they had decreased their leisure PA levels even though this was not the case.

Previous studies have shown that increased financial difficulties or economic worries due to the pandemic situation were associated with decreased PA and increased alcohol consumption [58, 59]. However, in the present study our results did not change when adjusting for the impact of the pandemic on the private financial situation.

A strength of this study is the longitudinal cohort design complemented with retrospective questions that enables measuring change both using a longitudinal change measure and a reported change measure. Another strength is the comprehensive design of the LSH research program, which includes multiple health risk behaviors and a broad range of variables capturing life conditions and change in life conditions. The population-based sample, with additional socioeconomic variables linked from registers, made it possible to adjust analyses for possible confounders. As all subgroups in the study (defined by sex, age or SES) were represented by substantial numbers of respondents, our assessment is that results were not limited by power. The study population in the present study was from the county of Östergötland, which means that it cannot automatically be generalized to all of Sweden. However, the county of Östergötland has been shown to be representative to the national average in terms of health indicators and health behaviors [60]. A more serious threat against the generalizability of the results to the general population is the fact that the invitation to the study was coordinated with the participation in health-promoting health dialogues offered by the primary health care centers. As it has been recognized that the most vulnerable groups are not reached by health-promoting interventions to the same extent as other groups [61], this poses a risk that vulnerable populations are not enough represented in the sample. Therefore, the SES differences in change in health behavior found in the present study could well be underestimated. Another potential shortcoming is that reported change in leisure PA and alcohol consumption, respectively, were measured by single questions with only a limited number of response categories. This means that minor nuances in behavioral patterns and changes might have been missed. It also implies that we cannot determine whether, for instance, a decrease in leisure PA poses a risk for deterioration of health status. Also, an aspect that calls for caution in interpretation of results based on longitudinal change measure is that the questionnaires at baseline was accompanied by a health dialogue, whereas the COVID questionnaire did not involve any personal interaction with health care representatives. This implies a risk that responses at baseline were more biased towards social desirability. This may especially apply to questions targeting alcohol consumption. However, the proportion of respondents that reported a pattern of risky alcohol consumption was still higher at baseline, compared with during the pandemic. If a social desirability bias existed, the actual decrease during the pandemic in regard to reported alcohol consumption would likely be even more pronounced than in our current findings. Finally, the sample only consists of individuals 40 years and older at baseline, which means that our findings cannot be generalized to the general adult Swedish population.

## Conclusion

Based on data from a longitudinal cohort study in Sweden, we found that most individuals did not change either their leisure PA levels or their alcohol consumption during the pandemic. However, a decrease in leisure PA was more common than an increase, and females were more likely to change their leisure PA levels during the pandemic. The most profound decreases in leisure PA were observed in individuals of working age. It was common to decrease the alcohol consumption than to increase it, but individuals aged 40 or 45 at baseline were more likely than other respondents to initiate a risky pattern of alcohol consumption. In conclusion, societal restrictions during a pandemic pose a risk for decreased leisure PA, especially among individuals in working age. However, such restrictions and altered life circumstances can seemingly also open a window of opportunity to increase leisure PA levels, not least for females. In addition, in future pandemics targeted interventions to prevent vulnerable groups from developing a risky alcohol consumption or of becoming physically inactive should be considered.

### Electronic supplementary material

Below is the link to the electronic supplementary material.

Additional file 1: Results of multinomial logistic regression. Longitudinal change in leisure PA by population groups.

Additional file 2: Results of multinomial logistic regression. Measured change in alcohol consumption by population groups.

Additional file 3: Results of multinomial logistic regression. Reported change in PA by population groups.

Additional file 4: Results of multinomial logistic regression. Reported change in alcohol consumption by population groups.

Additional file 5: Baseline levels of leisure PA by SES

## Data Availability

The datasets generated and analyzed during the current study are not publicly available due to privacy reasons but are available from the corresponding author on reasonable request.

## Abbreviations

COVID-19: Sars-Cov-2 coronavirus disease 2019
CES-D: Center for Epidemiologic Studies Depression Scale
CI: Confidence interval
LSH: Life conditions, stress and health study
OR: Odds ratio
PA: Physical activity
Sars-Cov-2: Severe acute respiratory syndrome coronavirus 2
SES: Socioeconomic status
SOC: Sense of coherence
WHO: The World Health Organization
