# Supplementary material for: Impact of the pandemic on leisure physical activity and alcohol consumption
Source: BMC Public Health. 2024 Jun 13;24:1589. doi: 10.1186/s12889-024-19100-w (PMC11177532; doi:10.1186/s12889-024-19100-w)
Supplement: Supplementary file 1 — Additional file 1: Results of multinomial logistic regression. Longitudinal change in leisure PA by population groups. [file 12889_2024_19100_MOESM1_ESM.pdf]

Additional file 2. Results of multinomial logistic regression. Longitudinal change in alcohol consumption by population groups

		Change in alcohol consumption	Model 1 (adjusted for alcohol consumption at baseline, prevalence of severe health problems, sex, and age)			Model 2 (model 1 + adjusted for cohabitation status and change in financial situation)		
Sex			n=2307	OR	95 % CI	n=2215	OR	95 % CI
	Males (reference)							
	Females	Unchanged		ref			ref	
		Change from risk consumption to consumption not considered risky		0.5	0.4–0.7		0.5	0.4–0.7
		Change from consumption not considered risky to risky consumption		0.8	0.6–1.1		0.8	0.6–1.2
Age			n=2307	OR	95 % CI	n=2215	OR	95 % CI
	40 years	Unchanged		ref			ref	
		Change from risk consumption to consumption not considered risky		2.1	1.3–3.5		2.2	1.3–3.6
		Change from consumption not considered risky to risky consumption		3.0	1.6–5.7		2.6	1.3–5.1
	45 years	Unchanged		ref			ref	
		Change from risk consumption to consumption not considered risky		1.7	1.1–2.7		1.7	1.1–2.8
		Change from consumption not considered risky to risky consumption		1.8	0.9–3.4		1.7	0.8–3.3
	50 years	Unchanged		ref			ref	
		Change from risk consumption to consumption not considered risky		1.5	1.0–2.4		1.5	1.0–2.4
		Change from consumption not considered risky to risky consumption		1.3	0.7–2.5		1.0	0.5–2.0
	55 years	Unchanged		ref			ref	
		Change from risk consumption to consumption not considered risky		1.4	0.9–2.1		1.5	1.0–2.3
		Change from consumption not considered risky to risky consumption		1.0	0.5–2.0		1.0	0.5–2.0
	60 years	Unchanged		ref			ref	
		Change from risk consumption to consumption not considered risky		1.5	1.0–2.1		1.5	1.0–2.2
		Change from consumption not considered risky to risky consumption		1.5	0.9–2.6		1.4	0.8–2.5
	65 years (reference)							
	70 years	Unchanged		ref			ref	

		Change from risk consumption to consumption not considered risky		0.9	0.7–1.4		0.9	0.6–1.4
		Change from consumption not considered risky to risky consumption		1.4	0.8–2.4		1.3	0.7–2.3
Educational level	Compulsory	Unchanged	n=2302	ref		n=2210	ref	
		Change from risk consumption to consumption not considered risky		1.0	0.7–1.6		1.0	0.6–1.6
		Change from consumption not considered risky to risky consumption		1.4	0.7–2.5		1.1	0.6–2.2
	Secondary school 2 years	Unchanged		ref			ref	
		Change from risk consumption to consumption not considered risky		1.3	1.0–1.9		1.4	1.0–1.9
		Change from consumption not considered risky to risky consumption		1.6	1.0–2.6		1.6	1.0–2.6
	Secondary school 3 years	Unchanged		ref			ref	
		Change from risk consumption to consumption not considered risky		1.2	0.8–1.7		1.1	0.8–1.7
		Change from consumption not considered risky to risky consumption		0.7	0.4–1.3		0.6	0.3–1.2
	Post-secondary school 3 years	Unchanged		ref			ref	
		Change from risk consumption to consumption not considered risky		1.1	0.8–1.6		1.1	0.8–1.7
		Change from consumption not considered risky to risky consumption		0.9	0.5–1.5		0.9	0.5–1.5
	Post-secondary school more than 3 years (reference)							
Household income	Q1 (lowest)	Unchanged	n=2303	ref		n=2211	ref	
		Change from risk consumption to consumption not considered risky		1.0	0.7–1.3		1.0	0.7–1.4
		Change from consumption not considered risky to risky consumption		1.1	0.7–1.7		1.2	0.7–1.9
	Q2	Unchanged		ref			ref	

		Change from risk consumption to consumption not considered risky		0.9	0.7–1.3		0.9	0.7–1.3
		Change from consumption not considered risky to risky consumption		0.8	0.5–1.3		0.8	0.5–1.3
	Q3	Unchanged		ref			ref	
		Change from risk consumption to consumption not considered risky		1.2	0.9–1.6		1.2	0.8–1.6
		Change from consumption not considered risky to risky consumption		0.7	0.4–1.2		0.7	0.4–1.2
	Q4 (reference)							
