# Supplementary material for: Impact of the pandemic on leisure physical activity and alcohol consumption
Source: BMC Public Health. 2024 Jun 13;24:1589. doi: 10.1186/s12889-024-19100-w (PMC11177532; doi:10.1186/s12889-024-19100-w)
Supplement: Supplementary file 2 — Additional file 2: Results of multinomial logistic regression. Measured change in alcohol consumption by population groups. [file 12889_2024_19100_MOESM2_ESM.pdf]

Additional file 1. Results of logistic regression. Longitudinal change in leisure PA by population groups

		Change in leisure PA	Model 1 (adjusted for leisure PA at baseline, prevalence of severe health problems, sex, and age)			Model 2 (model 1 + adjusted for cohabitation status and change in financial situation)		
Sex			n=2343	OR	95 % CI	n=2241	OR	95 % CI
	Males (reference)							
	Females	Unchanged		ref			ref	
		Increase		0.7	0.5–0.9		0.7	0.5–0.9
		Decrease		1.5	1.1–1.9		1.5	1.2–1.9
Age			n=2343	OR	95 % CI	n=2241	OR	95 % CI
	40 years	Unchanged		ref			ref	
		Increase		2.9	1.8–4.6		3.1	1.9–5.0
		Decrease		0.5	0.3–0.8		0.5	0.3–0.8
	45 years	Unchanged		ref			ref	
		Increase		2.4	1.5–3.9		2.6	1.6–4.2
		Decrease		0.7	0.5–1.2		0.7	0.5–1.1
	50 years	Unchanged		ref			ref	
		Increase		1.7	1.1–2.8		1.9	1.2–3.1
		Decrease		0.9	0.5–1.3		0.8	0.5–1.3
	55 years	Unchanged		ref			ref	
		Increase		1.3	0.9–2.1		1.4	0.9–2.2
		Decrease		0.9	0.6–1.5		0.9	0.6–1.4
	60 years	Unchanged		ref			ref	
		Increase		1.3	0.8–1.9		1.4	0.9–2.2
		Decrease		0.8	0.5–1.3		0.8	0.5–1.2
	65 years (reference)							
	70 years	Unchanged		ref			ref	
		Increase		0.4	0.2–0.7		0.5	0.3–0.8
		Decrease		1.7	1.1–2.5		1.7	1.1–2.6
Educational level	Compulsory	Unchanged	n=2339	ref		n=2237	ref	
		Increase		0.8	0.5–1.3		0.9	0.5–1.4
		Decrease		1.8	1.1–2.8		1.8	1.1–2.9
	Secondary school 2 years	Unchanged		ref			ref	
		Increase		0.7	0.5–1.0		0.6	0.5–0.9
		Decrease		1.7	1.2–2.3		1.8	1.2–2.5
	Secondary school 3 years	Unchanged		ref			ref	
		Increase		0.7	0.4–1.0		0.6	0.4–1.0
		Decrease		1.1	0.7–1.6		1.2	0.8–1.8
	Post-secondary school 3 years	Unchanged		ref			ref	
		Increase		1.0	0.7–1.4		0.9	0.6–1.3
		Decrease		1.6	1.1–2.2		1.6	1.1–2.3
	Post-secondary school more than 3 years (reference)							
Household income	Q1 (lowest)	Unchanged	n=2340	ref		n=2238	ref	
		Increase		0.9	0.6–1.3		0.8	0.5–1.2
		Decrease		2.2	1.5–3.3		2.2	1.4–3.3
	Q2	Unchanged		ref			ref	
		Increase		1.0	0.7–1.6		1.0	0.7–1.5
		Decrease		1.7	1.2–2.4		1.7	1.2–2.4
	Q3	Unchanged		ref			ref	
		Increase		0.9	0.7–1.4		0.9	0.6–1.3
		Decrease		1.3	0.9–1.9		1.4	1.0–2.0
	Q4 (reference)							
