# Supplementary material for: Impact of the pandemic on leisure physical activity and alcohol consumption
Source: BMC Public Health. 2024 Jun 13;24:1589. doi: 10.1186/s12889-024-19100-w (PMC11177532; doi:10.1186/s12889-024-19100-w)
Supplement: Supplementary file 3 — Additional file 3: Results of multinomial logistic regression. Reported change in PA by population groups. [file 12889_2024_19100_MOESM3_ESM.pdf]

Additional file 4. Results of multinomial logistic regression. Reported change in alcohol consumption by population groups

		Change in alcohol consumption	Model 1 (adjusted for prevalence of severe health problems, sex, and age)			Model 2 (model 1 + adjusted for cohabitation status and change in financial situation)		
Sex			n=2419	OR	95 % CI	n=2310	OR	95 % CI
	Males (reference)							
	Females	Unchanged		ref			ref	
		Decrease		1.0	0.8–1.3		1.0	0.8–1.3
		Increase		0.9	0.6–1.1		0.9	0.7–1.2
Age			n=2419	OR	95 % CI	n=2310	OR	95 % CI
	40 years	Unchanged		ref			ref	
		Decrease		2.3	1.5–3.7		2.2	1.4–3.5
		Increase		2.0	1.2–3.4		1.8	1.1–3.0
	45 years	Unchanged		ref			ref	
		Decrease		1.8	1.2–2.8		1.8	1.1–2.7
		Increase		1.1	0.7–2.0		1.1	0.6–1.9
	50 years	Unchanged		ref			ref	
		Decrease		1.2	0.7–1.9		1.2	0.7–1.9
		Increase		0.9	0.5–1.6		0.8	0.5–1.5
	55 years	Unchanged		ref			ref	
		Decrease		1.2	0.8–1.9		1.1	0.7–1.8
		Increase		0.9	0.5–1.5		0.8	0.5–1.4
	60 years	Unchanged		ref			ref	
		Decrease		1.2	0.8–1.9		1.3	0.8–1.9
		Increase		1.0	0.6–1.6		1.0	0.6–1.6
	65 years (reference)							
	70 years	Unchanged		ref			ref	
		Decrease		1.2	0.8–1.9		1.3	0.8–2.0
		Increase		0.8	0.5–1.4		0.8	0.5–1.4
Educational level	Compulsory	Unchanged	n=2414	ref		n=2305	ref	
		Decrease		0.8	0.5–1.2		0.9	0.5–1.3
		Increase		0.7	0.4–1.2		0.7	0.4–1.3
	Secondary school 2 years	Unchanged		ref			ref	
		Decrease		0.6	0.4–0.9		0.6	0.5–0.9
		Increase		0.5	0.3–0.8		0.5	0.4–0.8
	Secondary school 3 years	Unchanged		ref			ref	
		Decrease		0.6	0.4–0.9		0.6	0.4–0.9
		Increase		0.8	0.5–1.2		0.8	0.5–1.2
	Post-secondary school 3 years	Unchanged		ref			ref	
		Decrease		0.9	0.6–1.3		0.9	0.7–1.3
		Increase		0.8	0.5–1.2		0.8	0.5–1.2
	Post-secondary school more than 3 years (reference)							
Household income	Q1 (lowest)	Unchanged	n=2415	ref		n=2306	ref	
		Decrease		0.8	0.6–1.2		0.8	0.5–1.1
		Increase		0.6	0.4–0.9		0.6	0.4–1.0
	Q2	Unchanged		ref			ref	
		Decrease		0.9	0.6–1.3		0.8	0.6–1.2
		Increase		0.6	0.4–0.9		0.6	0.4–0.9
	Q3	Unchanged		ref			ref	
		Decrease		1.0	0.7–1.3		0.9	0.7–1.3
		Increase		0.9	0.6–1.4		1.0	0.7–1.5
	Q4 (reference)							
