# Supplementary material for: Impact of the pandemic on leisure physical activity and alcohol consumption
Source: BMC Public Health. 2024 Jun 13;24:1589. doi: 10.1186/s12889-024-19100-w (PMC11177532; doi:10.1186/s12889-024-19100-w)
Supplement: Supplementary file 4 — Additional file 4: Results of multinomial logistic regression. Reported change in alcohol consumption by population groups. [file 12889_2024_19100_MOESM4_ESM.pdf]

Additional file 3. Results of multinomial logistic regression. Reported change in leisure PA by population groups

		Change in leisure PA	Model 1 (adjusted for leisure PA at baseline, prevalence of severe health problems, sex, and age)			Model 2 (model 1 + adjusted for cohabitation status and change in financial situation)		
Sex			n=2339	OR	95 % CI	n=2237	OR	95 % CI
	Males (reference)							
	Females	Unchanged		ref			ref	
		Increase		1.6	1.3–2.0		1.7	1.4–2.1
		Decrease		1.8	1.4–2.2		1.9	1.5–2.3
Age			n=2339	OR	95 % CI	n=2237	OR	95 % CI
	40 years	Unchanged		ref			ref	
		Increase		1.7	1.1–2.6		1.6	1.0–2.4
		Decrease		1.5	1.0–2.2		1.3	0.9–2.0
	45 years	Unchanged		ref			ref	
		Increase		1.5	1.0–2.2		1.4	0.9–2.1
		Decrease		1.6	1.1–2.4		1.5	1.0–2.2
	50 years	Unchanged		ref			ref	
		Increase		1.1	0.7–1.6		1.0	0.6–1.5
		Decrease		1.6	1.1–2.3		1.5	1.0–2.2
	55 years	Unchanged		ref			ref	
		Increase		1.3	0.9–1.8		1.1	0.8–1.8
		Decrease		1.5	1.1–2.2		1.5	1.0–2.1
	60 years	Unchanged		ref			ref	
		Increase		1.6	1.1–2.2		1.6	1.1–2.3
		Decrease		1.1	0.8–1.6		1.1	0.8–1.6
	65 years (reference)							
	70 years	Unchanged		ref			ref	
		Increase		0.8	0.6–1.2		0.8	0.5–1.2
		Decrease		1.2	0.8–1.7		1.2	0.8–1.7
Educational level	Compulsory	Unchanged	n=2335	ref		n=2233	ref	
		Increase		0.6	0.4–0.8		0.6	0.4–0.9
		Decrease		0.4	0.3–0.6		0.5	0.3–0.7
	Secondary school 2 years	Unchanged		ref			ref	
		Increase		0.4	0.3–0.6		0.4	0.3–0.6
		Decrease		0.5	0.4–0.6		0.5	0.4–0.6
	Secondary school 3 years	Unchanged		ref			ref	
		Increase		0.7	0.5–0.9		0.7	0.5–0.9
		Decrease		0.6	0.4–0.8		0.6	0.4–0.8
	Post-secondary school 3 years	Unchanged		ref			ref	
		Increase		0.7	0.5–1.0		0.7	0.5–1.0
		Decrease		0.7	0.6–1.0		0.8	0.6–1.0
	Post-secondary school more than 3 years (reference)							
Household income	Q1 (lowest)	Unchanged	n=2336	ref		n=2234	ref	
		Increase		0.6	0.4–0.8		0.5	0.4–0.8
		Decrease		0.8	0.6–1.0		0.7	0.5–1.0
	Q2	Unchanged		ref			ref	
		Increase		0.8	0.6–1.1		0.7	0.5–1.0
		Decrease		0.7	0.5–0.9		0.7	0.5–0.9
	Q3	Unchanged		ref			ref	
		Increase		0.8	0.6–1.1		0.8	0.6–1.1
		Decrease		0.9	0.7–1.2		0.9	0.7–1.3
	Q4 (reference)							
