# Supplementary material for: Impact of the pandemic on leisure physical activity and alcohol consumption
Source: BMC Public Health. 2024 Jun 13;24:1589. doi: 10.1186/s12889-024-19100-w (PMC11177532; doi:10.1186/s12889-024-19100-w)
Supplement: Supplementary file 5 — Additional file 5: Baseline levels of leisure PA by SES [file 12889_2024_19100_MOESM5_ESM.pdf]

Additional file 5. Baseline levels of PA by SES

		Leisure physical activity (PA) level at baseline					
		Sedentary leisure time		Moderate exercise		Vigorous exercise	
		N	%	N	%	N	%
Educational level	Compulsory	28	10.2	198	72.3	48	17.5
	Secondary school 2 years	59	9.0	439	66.8	159	24.2
	Secondary school 3 years	30	8.4	213	59.7	114	32.0
	Post-secondary school 3 years	34	7.9	240	56.1	154	36.0
	Post-secondary school more than 3 years	47	7.3	360	55.9	237	36.8
	Total	198	8.4	1450	61.4	712	30.1
Household income	Q1 (lowest)	64	10.9	414	70.4	110	18.7
	Q2	60	10.2	357	60.4	174	29.4
	Q3	42	7.0	357	59.8	198	33.2
	Q4 (highest)	32	5.5	322	55.0	231	39.5
	Total	198	8.4	1450	61.4	713	30.2
